# Spectrum of digoxin-induced ocular toxicity: a case report and literature review

**DOI:** 10.1186/s13104-015-1367-6

**Published:** 2015-08-23

**Authors:** Delphine Renard, Eve Rubli, Nathalie Voide, François-Xavier Borruat, Laura E. Rothuizen

**Affiliations:** Division of Clinical Pharmacology, University Hospital of Lausanne, Bugnon 17-01, 1011 Lausanne, Switzerland; Service de gériatrie et réadaptation gériatrique, CHUV, CUTR Sylvana, Ch. de Sylvana 10, 1066 Epalinges, Switzerland; Département d’ophtalmologie, Université de Lausanne, Fondation Asile des Aveugles, Hôpital ophtalmique Jules-Gonin, av. de France 15, 1000 Lausanne 7, Switzerland

**Keywords:** Digoxin, Drug intoxication, Vision impairment, Dyschromatopsia, Photopsia

## Abstract

**Background:**

Digoxin intoxication results in predominantly digestive, cardiac and neurological symptoms. This case is outstanding in that the intoxication occurred in a nonagenarian and induced severe, extensively documented visual symptoms as well as dysphagia and proprioceptive illusions. Moreover, it went undiagnosed for a whole month despite close medical follow-up, illustrating the difficulty in recognizing drug-induced effects in a polymorbid patient.

**Case presentation:**

Digoxin 0.25 mg qd for atrial fibrillation was prescribed to a 91-year-old woman with an estimated creatinine clearance of 18 ml/min. Over the following 2–3 weeks she developed nausea, vomiting and dysphagia, snowy and blurry vision, photopsia, dyschromatopsia, aggravated pre-existing formed visual hallucinations and proprioceptive illusions. She saw her family doctor twice and visited the eye clinic once until, 1 month after starting digoxin, she was admitted to the emergency room. Intoxication was confirmed by a serum digoxin level of 5.7 ng/ml (reference range 0.8–2 ng/ml). After stopping digoxin, general symptoms resolved in a few days, but visual complaints persisted. Examination by the ophthalmologist revealed decreased visual acuity in both eyes, 4/10 in the right eye (OD) and 5/10 in the left eye (OS), decreased color vision as demonstrated by a score of 1/13 in both eyes (OU) on Ishihara pseudoisochromatic plates, OS cataract, and dry age-related macular degeneration (ARMD). Computerized static perimetry showed non-specific diffuse alterations suggestive of either bilateral retinopathy or optic neuropathy. Full-field electroretinography (ERG) disclosed moderate diffuse rod and cone dysfunction and multifocal ERG revealed central loss of function OU. Visual symptoms progressively improved over the next 2 months, but multifocal ERG did not. The patient was finally discharged home after a 5 week hospital stay.

**Conclusion:**

This case is a reminder of a complication of digoxin treatment to be considered by any treating physician. If digoxin is prescribed in a vulnerable patient, close monitoring is mandatory. In general, when facing a new health problem in a polymorbid patient, it is crucial to elicit a complete history, with all recent drug changes and detailed complaints, and to include a drug adverse reaction in the differential diagnosis.

## Background

Digitalis glycosides’ long history in medical practice has been beautifully explored by English pharmacologist Jeff K. Aronson some 20 years ago. His in-depth study, centered on the work by 18th century English physician and scientist William Withering, shows that their narrow therapeutic margin has been recognized and dealt with for several thousand years [1].

Digoxin is the main form of digitalis medically used in Switzerland today. It is extracted from the leaves of the plant *Digitalis lanata*. It combines with and reversibly inhibits the cell membrane Na+/K+-ATPase, inducing an increase in intracellular calcium, intracellular sodium, and extracellular potassium. In the heart, this leads to an increase in myocardium’s strength of contraction and excitability, as well as to a decrease in conduction and depolarization velocity in the atrio-ventricular node. Current indications to digoxin are restricted to second line treatment for paroxystic or permanent atrial fibrillation (AF), second line treatment for symptomatic heart failure with rapid AF, or as a last resort for severe, symptomatic systolic heart failure (left ventricular ejection fraction ≤40 %) [2, 3].

Pharmacological properties of digoxin are as follows: bioavailability 70–80 %, distribution volume 5–7 l/kg, poor protein binding (25 %), time to peak concentration 1 h, time to peak effect 6 h after oral administration. Metabolism is negligible and elimination essentially occurs through glomerular filtration, with a small contribution of tubular secretion involving P-glycoprotein. Digoxin has a long half-life (40 h; 100 h and beyond in kidney disease).

Usual maintenance dosing is 0.25 mg qd. In kidney disease, empirical dose reductions are proposed accordingly, for example 50–75 % of usual dosing for a creatinine clearance (CrCl) of 50–100 ml/min according to the Cockroft-Gault formula, 30–50 % of usual dosing for a CrCl of 20–50 ml/min, and 20–30 % of usual dosing for a CrCl <20 ml/min. These are general guidelines only, as plasma creatinine is a poor indicator of kidney function in very ill or sarcopenic elderly people. Drug monitoring and scrupulous follow-up are mandatory in such cases.

Digoxin toxicity predominantly manifests as digestive or neurological symptoms. Cardiac symptoms are less frequent but are related to the seriousness of digoxin poisoning, the overall rate of mortality being estimated at 25 %. Out of range potassium, calcium or magnesium levels can modulate digoxin action. Neurologic symptoms are multifold, including headache, confusion, somnolence, fatigue, restlessness and visual symptoms. Elderly patients are more susceptible to digoxin toxicity for a variety of reasons including kidney disease, a decrease in muscular mass, frequent polypharmacy (diuretics and amiodarone being the most frequent at-risk co-medication), and poor adherence due to sensory and cognitive impairment [4].

The earliest report of digitalis-associated ocular toxicity we could easily access was published in 1925 in the *Boston Medical and Surgery Journal*, ancestor of the *New England Journal of Medicine* [5]. According to the authors: “Sensory disturbances associated with an overdose of digitalis […] are met with rarely, and even then may easily be overlooked simply because their meaning is not clear.” They proceed with reporting the illness of a 55-year-old woman who, after 1 year of regularly taking digitalis, “developed an attack of failing vision. Everything appeared yellow before her eyes.” Later on, “She was unable clearly to distinguish objects and persons at a distance, and everything seemed to be bathed in a very intense glaring white light […]. The air seemed filled with yellow snow, and the grass appeared distinctly blue in color. The sky appeared green. At times she had double vision, […] at times she saw T shaped objects in the sky or any blank surface.” The authors conclude: “It may be that the condition of yellow vision is more common than we suppose and that more careful questioning of patients with toxic manifestations of digitalis will reveal this fact”.

As geriatricians, ophthalmologists, and clinical pharmacologists, we recently cared for an elderly woman suffering from several visual symptoms related to digoxin intoxication. We tell her story as a well documented case of digoxin ocular toxicity, as well as a reminder that the need for “careful questioning” still holds true in medical practice today.

## Case presentation

A 91-year-old female patient, weight 56 kg, height 151 cm, was admitted to the emergency room (ER) of a tertiary hospital complaining about dysphagia, vomiting and blurry vision. She was in altered general condition but hemodynamically stable; detailed physical examination was unremarkable. Electrocardiography showed paced rhythm, 60/min. She felt so ill she repeatedly said she’d have to enter a nursing home.

The patient lived alone at home. She got daily help from her daughter and the local home care services, and sometimes made mistakes while managing her medication. She suffered from multiple co morbidities, including hypertension, hypercholesterolemia, type 2 diabetes, ischemic heart disease with heart failure, chronic kidney disease (creatinine clearance according to Cockroft-Gault formula, CrCl, 18 ml/min), gout, and indolent breast cancer. A pacemaker had been implanted 2 years earlier for permanent atrial fibrillation (AF) with insufficiently controlled ventricular rate. She had benefited from right eye cataract surgery. She was under the regular care of a general practitioner (GP), a nephrologist, a cardiologist, an ophthalmologist and an oncologist.

Her medication consisted of acenocoumarol qd (dosing according to International Normalized Ratio, targeted range 2.0–3.0), metoprolol 100 mg qd, lisinopril 5 mg qd, torasemide 20 mg qd, simvastatin 20 mg qd, anastrozole 1 mg qd, ibandronate 150 mg monthly, lorazepam 0.5 mg qd, folate 5 mg qd, calcium 500 mg/cholecalciferol 400 IU bd, and zolpidem 5 mg qd as needed.

Digoxin, 0.25 mg qd, and diltiazem, 90 mg qd, had been started exactly 1 month earlier by her cardiologist for AF with rapid ventricular response. In a letter to the GP sent 7 days after the patient was started on digoxin, the cardiologist mentioned the need for measuring the digoxin level and adapting the dosing, without specifying a deadline. He also reported intending atrioventricular node ablation therapy 5 weeks later.

A detailed history given by the patient, her daughter, and the home care services revealed that on the 7th day after starting digoxin, the patient suffered a fall, which led her GP to stop diltiazem on the 8th day. On the 13th day, she had “digestive symptoms” and difficulty eating. From the 18th day on, she complained of significant loss of vision in both eyes, seeing “everything in white”.

Five months previously, she had been routinely examined by her ophthalmologist. Visual acuity was 10/10 difficult in both eyes, and fundus examination disclosed the presence of slight retinal pigment epithelium changes compatible with early dry age-related macular degeneration (ARMD). On the 21th day of digoxin therapy, an emergency consultation at the eye clinic revealed decreased visual acuity in both eyes (4/10 OD and 5/10 OS), a mature cataract in the left eye and dry ARMD in both eyes. From then on, the patient lost 5 kg because of nausea and vomiting, and her vision remained severely impaired.

Her symptoms varied from day to day but included blurry vision (she couldn’t read a printed text anymore), snowy vision (everything appeared to be bathed in a white light), a tendency to collide with objects on her right side, dyschromatopsia (she was seeing blue or purple spots when she closed her eyes, vivid colors appeared to be faded), formed visual hallucinations of little human figures that did not frighten her appearing either upon awakening (hypnopompic) or shortly before falling asleep (hypnagogic), and feeling as if she was on a fishing boat when lying in bed (proprioceptive illusions).

Digoxin intoxication was suspected on admission 1 month after its initiation, and confirmed when the serum level was measured to be 5.7 ng/ml [0.8–2.0].^1^ Other relevant laboratory results were as follows (reference range in brackets): creatinine 183 µmol/l [44–80], sodium 144 mmol/l [135–145], potassium 4.0 mmol/l [3.5–4.6], calcium 2.41 mmol/l [2.10–2.50].

Digoxin was definitively stopped, and further levels were as follows (all samples were taken at 6 am, day 0 = day of ER admission): Day 3: 4.0 ng/ml. Day 10: 1.1 ng/ml. Day 18: not to be detected. On this basis, we estimated the half-life to be approximately 80–90 h in this patient at that time. Chest radiography, brain computed tomography scan and oesogastric studies provided no contributive finding in explaining the symptoms.

After digoxin withdrawal, nausea and vomiting were first to disappear in the next few days. Her general condition then improved, the patient gained weight and benefited from a hospital rehabilitation program.

On the 17th day after ER admission, a detailed ophthalmological examination was performed. Visual symptoms were persistent, including snowy and blurry vision, peripheral fixed photopsias and dyschromatopsia, all compatible with digoxin intoxication, as well as formed visual hallucinations occurring systematically when the patient fell asleep (hypnagogic visual hallucinations). Visual acuity was decreased in both eyes (4/10 OD with S−1.25, C−1.5 at 103° and 5/10 OS with S+1.25) and color vision testing with Ishihara pseudoisochromatic plates disclosed a severe red-green dyschromatopsia (only 1/13 plates correctly identified with either eye). Slit-lamp examination showed a pseudophakic OD and a mature corticonuclear OS cataract. Intraocular pressures were normal (12 mmHg OU). Funduscopy revealed normal optic nerve head with peripapillary atrophy OU, normal retinal vessels and bilateral minimal macular changes compatible with bilateral early dry ARMD. Computerised static perimetry (Octopus model 300) showed non-specific diffuse sensitivity loss consistent with bilateral diffuse retinopathy. Compared to our normative data for the age group, full-field photopic and scotopic ERGs confirmed a moderate diffuse dysfunction more pronounced for cones than rods (photopic and scotopic b-wave amplitude was decreased to 60 % of the lower normal values for the age). Qualitative interpretation of multifocal ERG disclosed a diffuse decrease of amplitude mostly in the central ten degrees.

The patient was finally discharged home 44 days after ER admission. She was still living at home 6 months after ER admission according to elicited follow-up. At that time, tests showed a subtle improvement of visual acuity (6/10 OD and 5/10 OS), persistent severe red-green dyschromatopsia (1/13 on Ishihara test OU), and a slight non significant amplitude increase on the multifocal ERG. Unfortunately, she denied further ophthalmologic examinations beyond follow-up at 6 months.

## Discussion and literature review

In this patient, the time course and characteristics of the clinical evolution, and the constellation of symptoms were attributed with high probability to digoxin intoxication, notably in view of the elevated drug level (score of 9/13 according to the Naranjo Adverse Drug Reaction Probability Scale [6]).

Many risk factors for these adverse drug reactions were present:

1. A drug with a narrow therapeutic margin was administered at standard dosing with insufficient pre-defined follow-up strategy to a particularly vulnerable patient because of advanced age, polymorbidity, polymedication with questionable adherence, and pre-existing advanced kidney disease as well as functional and ocular impairment. Specific interacting drugs were diltiazem (which increases exposure to digoxin through uncertain mechanisms, possibly by inhibiting P-glycoprotein) and torasemide and lisinopril (which aggravate renal failure and the risk of dehydration and electrolyte imbalance).
2. Many people were involved in the care of the patient, and her symptoms were, at least in the beginning, entirely non-specific. Loose communication between the cardiologist and the GP, dilution of responsibilities and failure to rapidly identify and incriminate drugs as a plausible cause were all elements that could contribute to the delay in diagnosing this potentially life-threatening intoxication.

Further discussion will focus on neurologic symptoms, with particular emphasis on visual symptoms.

Although dysphagia was mentioned on admission, it is difficult to be positive it was really part of the clinical picture, because the patient then stopped complaining about it and later even denied having ever suffered from it. Two case reports describe dysphagia in digoxin intoxication in elderly women, but the clinical picture was more clear-cut [7, 8].

Proprioceptive illusions are described in another case report as “[the patient complained that] the room was hilly and that the bed was continuously sliding downhill” [9].

Disturbances of vision during digoxin treatment are less common than cardiac or other non cardiac symptoms, but are considered by some authors to be more specific. In this patient, cardiac symptoms are likely to have been concealed by the implanted pacemaker. The spectrum of visual complaints include decreased visual acuity, central scotomas or visual field reduction, glare, photopsia most pronounced in daylight, photophobia, blurry or snowy vision, visual hallucinations, diplopia, and dyschromatopsia including xanthopsia (yellow vision), cyanopsia (blue vision) and chloropsia (green vision). Dyschromatopsia can be asymptomatic and revealed only by formal testing; several studies report a positive correlation between the total error score at testing and serum digoxin level. A more recent study in 30 elderly hospitalized patients receiving digoxin maintenance therapy compared to controls did not confirm this correlation, but the authors reported a high incidence of impaired color vision at serum digoxin levels considered therapeutic as well as supra-therapeutic, speaking for limited value of formal color vision testing for the detection of toxicity in the aged [10].

The mechanism underlying ocular symptoms is presumed to be related to Na+ K+ATPase inhibition but remains speculative [9, 11]. Ophthalmologic tests considered most useful to support a diagnosis of digoxin intoxication are photopic and scotopic ERG seeking for b-wave delayed implicit time and decreased b-wave amplitude.

In our patient, visual symptoms improved after stopping digoxin, yet some persisted for several weeks, long after drug levels became undetectable, and others had not resolved after 6 months. We offer several possible explanations for this. First, digoxin elimination was slow because of its high volume of distribution and prolonged half-life in this patient. It could also be hypothesized that serum digoxin levels more poorly correlate with certain specific pharmacodynamic effects (for example, duration of the effect may possibly vary according to different clearances from deep tissue compartments or variability in inhibition by digoxin of different Na /K+-ATPase isoforms in different body tissues). Second, the patient may have been more worried about and inclined to express ocular symptoms because of the attention given to them by clinicians. Third, the underlying eye diseases may themselves have evolved over the time. Eventually, the patient refused to continue ophthalmologic follow-up beyond 6 months hereby escaping longer monitoring.

Arbitrarily starting our literature review in the seventies, we found a total of 15 references to case reports of impaired vision during digoxin maintenance therapy or in situations of chronic supratherapeutic digoxin levels. These publications describe a total of 52 patients (Table 1), with only five cases described in more recent years (since 2000). Quality of documentation and degree of detail are highly unequal among studies, which does not allow for a quantitative analysis. As a whole, most patients are aged (>65-year-old), with only one patient being under 40. Ocular symptoms are numerous. Digoxin levels also vary greatly, but many are in the supratherapeutic or more clearly toxic range. Finally, full resolution is almost always witnessed over the course of 1–2 weeks, but here again follow-up is heterogeneous and often of short duration.

**Table 1:** Synopsis of cases of visual symptoms in digoxin maintenance therapy or digoxin chronic intoxication published in Pubmed, 1974–2006

References	Patient(s) (F = female or M = male, age in years)	Digoxin treatment (mg qd unless otherwise specified)	Treatment duration	Ocular symptoms	Precipitating factors	Digoxin level (ng/ml)	Specific ophthalmologic examination	Evolution after stopping digoxin (or, in a few cases, significantly decreasing the dose)
Manninen [12]	n = 5 2 M, 3 F, 61–78	0.15–0.75	Several years	Indistinct vision of objects	None detected	1.3 (n = 1) 2.1–3.2 (n = 4)	Ishihara plates: “some degree of difficulty in seeing the plates for green–red vision”^b^	Full resolution at 2 weeks
Volpe and Soave [13]	n = 3 2 M, 1 F, 66–82	^a^	^a^	Yellow hue to objects, formed visual hallucinations (animals, people, household objects)	a) ^a^ b) Dose increase to 0.375 mg bd c) ^a^	a) 5.0 b) 6.5 c) 2.7	Not mentioned^b^	Full resolution after drug level corrected
Aronson and Ford [11]	n = 10 (experiment on subjects previously published)	Not specified in this article	Not specified in this article	–	Not specified in this article	Not specified in this article	Farnsworth-Munsell 100-Hue test (upper limit of normal for colour defect score = 140). “The median colour defect score in toxic patients (212) was significantly higher than that of controls (85), falling to the significantly lower value of 127 after withdrawal of digoxin […]	Full resolution
Weleber and Shults [14]	M, 70	0.25 four times daily	At least several months	Glare sensitivity, dimming of vision, silver flashes in the form of rectangles in the right visual field, halos around lights, no yellow vision	Concomitant quinidine sulfate administration	3.5	Farnsworth-Munsell 100-Hue error scores: 569 OD, 646 OS Electrooculogram: high initial light-to-dark ratios (3.50 OD, 3.20 OS) Electroretinogram: No detectable dark-adapted cone-mediated response (x wave). Light-adapted cone-mediated response (b wave) significantly subnormal and significantly prolonged in implicit time. Dark-adapted rod-mediated b wave significantly subnormal and prolonged in implicit time. Amplitude of the scotopic a wave (corneal negative peak) and bx wave (corneal positive peak) at maximal stimulus intensity subnormal.	Full resolution after 2 weeks
Closson [9]	F, 84	0.5	38 months	Blue, green and yellow veils floating everywhere, pleasant visions of trees, sun, etc. No color vision impairment.	Concomitant hydrochlorothiazide and triamterene prescription for 2 days	2.2	^a^	Full resolution
Chuman and LeSage [15]	n = 2 a) M, 79 b) M, 61	a) 0.25 and 0.125 on alternate days b) 0.125	^a^	a) “Objects appearing more white than usual” b) Objects were seen as if they were in a fog and colors appeared to be faded	^a^	a) 13.1 b) 6	Farnsworth-Munsell 100-Hue test error score: a) 497 b) 854 Ishihara plates: a) 7 errors/failed b) 14 errors/failed AOH-R-R plate defects: a) medium for green–red and blue-yellow b) strong for red-green and blue-yellow	^a^
Merté et al. [16]	n = 14, median age 72 years (prospective study comparing subjects with digoxin and digitoxin intoxication)	^a^	At least 6 weeks	One-third of patients complained about blurry vision, photopsia and impairment of color vision	^a^	Median digoxin level 2.98 (2.49–5.4)	Farnsworth-Munsell 100-Hue test: median number of errors 170	Significant improvement, follow-up restricted to 8 days
Hobley and Lawrenson [17]	F, 36	0.25	3 months	Scintillating visual field loss, in attacks each lasting about 20 min. No alteration in color vision	^a^	<2	Friedman Visual Field Analyser Mark II: relative defect of visual field between 15 and 20 degrees in temporal field OD, and between 15 and 20 nasal field OS Farnsworth-Munsell 100-Hue error score: 20 OD, 84 OS Ishihara plates: results normal OU	Full resolution
Piltz et al. [18]	n = 3 a) F, 88 b) F, 78 c) F, 58 (with retinitis pigmentosa and high myopia)	a) 0.25 b) 0.25 c) 0.25	a) ^a^ b) 2 months c) ^a^	a) Progressive, generalized “dimming” of vision OU b) decreased visual acuity bilaterally with snowy vision, obscuring vision of colors c) “extreme darkness of vision”	^a^	a) 6.1 b) 2.2 c) 4.5	Electroretinogram (ERG) a) amplitude of dark adapted flash ERG decreased to ~ 20 % of normal OD, and 45 % of normal OS Goldman perimetry b) generalized depression of the field and 10-degree relative central scotoma to the I3E isopter OS Ishihara plates b) correctly identified only the test plate (not all tests available for all patients; no specific tests mentioned for patient c)	a) Partial resolution only at 3 months b) Full resolution at 1 month c) Full resolution
Butler et al. [19]	n = 6 a) F, 76 b) F, 79 c) F, 82 d) M, 75 e) F, 85 f) M, 66	a) 0.25 b) 0.25–0.75 c) 0.25 d) 0.375 e) 0.25 f) 0.25	^a^	a) “Light through vertical blinds” b) Flashing lights temporally c) Yellow and white sparks dancing and swirling over whole visual field d) Flickering in periphery of vision e) Glare, brightness OD f) White glare, “snow on grass, trees”, decreased visual acuity and color discrimination	^a^	a) 0.5–1.4 b) 0.5–0.9 c) 0.8 d) 1.4–1.5 e) 0.7 f) 0.2	Electroretinogram cone b-wave explicit time [ms]: a) 30.0 OD, 28.8 OS b) 40 OD c) not done d) 37.6^b^ e) 35.4^b^ f) 36.4 OD, 36.0 OS	Full resolution
Wolin [20]	n = 2 a) F, 68 b) F, 63	^a^	^a^	a) Shimmering lights b) Blurred vision OU	^a^	a) 1.7 b) 1.0	Humphrey automated visual field testing and fluorescein angiography: a) not mentioned b) generalized depression OU with greater inferior than superior visual field loss^b^	a) Full resolution in 2 weeks b) Partial resolution
Honrubia et al. [21]	M, 72	^a^	2 months	Photopsia in the whole visual field	^a^	2.75	Humphrey automated visual field testing: normal (81 points) Farnsworth-Munsell 100-Hue test: normal	Significant improvement after 3 days
Nagai et al. [22]	n = 2 a) M, 72 b) M, 54	^a^	^a^	Severe decrease in visual acuity	^a^	^a^	Electroretinogram (ERG): 30 Herz-flicker ERG and photopic ERG: decreased amplitude^b^	^a^
Gödecke-Koch et al. [23]	F, 90 (severe eye disease after bilateral zoster infection)	0.2	^a^	Visual hallucinations (people, yellow hay, colored surfaces)	^a^	1.9	^a^	Full resolution
Oishi et al. [24]	F, 72	0.125	1 year	Sudden onset of binocular photopsia in the periphery of visual fields	Diarrhea with dehydration and hyperkaliemia	1.7	^a^	Full resolution in 2 weeks

*OD* right eye, *OS* left eye, *OU* both eyes

^a^No information available

^b^No further details or quantification available

## Conclusion

We describe a case of digoxin intoxication in a patient at highest risk for this adverse outcome due to different endogenous and exogenous factors: advanced age, polymorbidity including severe chronic kidney disease, preexistent ocular disease, insufficient communication among carers and failure to include a drug-induced adverse reaction in the differential diagnosis. It is unique by its extensive description of ophthalmologic findings, over a 6 month follow-up. Above all, this case is a reminder of a complication of digoxin treatment to be considered by any treating physician. If digoxin is prescribed in a vulnerable patient, close monitoring is mandatory. In general, when facing a new health problem in a polymorbid patient, it is crucial to elicit a complete history, with all recent drug changes and detailed complaints, and to include a drug adverse reaction in the differential diagnosis.

## Consent

Written informed consent was obtained from the patient for publication of this case report. A copy of the written consent is available for review by the Editor-in-Chief of this journal.

## Footnotes

1 The last time the patient had taken digoxin could not be determined with certainty, but was supposed to have been approximately 48 h prior to blood sampling.
